# Excitotoxic targeting of Kidins220 to the Golgi apparatus precedes calpain cleavage of Rap1-activation complexes

**DOI:** 10.1038/s41419-019-1766-z

**Published:** 2019-07-11

**Authors:** Celia López-Menéndez, Ana Simón-García, Andrea Gamir-Morralla, Julia Pose-Utrilla, Rafael Luján, Naoki Mochizuki, Margarita Díaz-Guerra, Teresa Iglesias

**Affiliations:** 1 Instituto de Investigaciones Biomédicas “Alberto Sols”, Consejo Superior de Investigaciones Científicas-Universidad Autónoma de Madrid (CSIC-UAM), C/ Arturo Duperier, 4, 28029 Madrid, Spain; 2 Centro de Investigación Biomédica en Red de Enfermedades Neurodegenerativas (CIBERNED), Instituto de Salud Carlos III, C/ Valderrebollo, 5, 28031 Madrid, Spain; 3 Synaptic Structure Laboratory, Instituto de Investigación en Discapacidades Neurológicas (IDINE), Dept. Ciencias Médicas, Facultad de Medicina, Universidad Castilla-La Mancha, Campus Biosanitario, C/ Almansa 14, 02008 Albacete, Spain; 4 Department of Cell Biology, National Cerebral and Cardiovascular Center Research Institute, 5-7-1 Fujishiro-dai, Suita, 565-8565 Osaka Japan; 5 Present Address: Institute of Physiological Chemistry, University Medical Center, Johannes Gutenberg University Mainz, Hanns-Dieter-Hüsch-Weg 19, 55128 Mainz, Germany

**Keywords:** Mechanisms of disease, Endocytosis, Cellular neuroscience, Ion channels in the nervous system, Stroke

## Abstract

Excitotoxic neuronal death induced by high concentrations of glutamate is a pathological event common to multiple acute or chronic neurodegenerative diseases. Excitotoxicity is mediated through overactivation of the N-Methyl-D-aspartate type of ionotropic glutamate receptors (NMDARs). Physiological stimulation of NMDARs triggers their endocytosis from the neuronal surface, inducing synaptic activity and survival. However almost nothing is known about the internalization of overactivated NMDARs and their interacting proteins, and how this endocytic process is connected with neuronal death has been poorly explored. Kinase D-interacting substrate of 220 kDa (Kidins220), also known as ankyrin repeat-rich membrane spanning (ARMS), is a component of NMDAR complexes essential for neuronal viability by the control of ERK activation. Here we have investigated Kidins220 endocytosis induced by NMDAR overstimulation and the participation of this internalization step in the molecular mechanisms of excitotoxicity. We show that excitotoxicity induces Kidins220 and GluN1 traffic to the Golgi apparatus (GA) before Kidins220 is degraded by the protease calpain. We also find that excitotoxicity triggers an early activation of Rap1-GTPase followed by its inactivation. Kidins220 excitotoxic endocytosis and subsequent calpain-mediated downregulation governs this late inactivation of Rap1 that is associated to decreases in ERK activity preceding neuronal death. Furthermore, we identify the molecular mechanisms involved in the excitotoxic shutoff of Kidins220/Rap1/ERK prosurvival cascade that depends on calpain processing of Rap1-activation complexes. Our data fit in a model where Kidins220 targeting to the GA during early excitotoxicity would facilitate Rap1 activation and subsequent stimulation of ERK. At later times, activation of Golgi-associated calpain, would promote the degradation of GA-targeted Kidins220 and two additional components of the specific Rap1 activation complex, PDZ-GEF1, and S-SCAM. In this way, late excitotoxicity would turn off Rap1/ERK cascade and compromise neuronal survival.

## Introduction

Overstimulation of the N-Methyl-D-aspartate type of ionotropic glutamate receptors (NMDARs) triggers excitotoxicity, a type of neuronal death that contributes to neurodegeneration in acute neuropathologies (stroke, traumatic brain injury, and epilepsy) and chronic neurodegenerative diseases (Alzheimer’s, Parkinson’s, or Huntington’s)^1^. By contrast, activation of NMDARs with physiological concentrations of glutamate is fundamental to neuronal synaptic activity, playing key roles in plasticity, learning and memory^2^, as well as neuronal survival through activation of extracellular signal-regulated kinase (ERK)^3^.

Functional NMDARs are heteromeric complexes composed primarily of two obligatory GluN1 subunits and two GluN2 or GluN3 subunits^4^. Diversity in NMDARs properties is mainly conferred by GluN2A and GluN2B subunits^4^. Function and signaling of NMDARs relie on their spatial and temporal distribution at the neuronal surface where they undergo endocytosis after exposure to nonpathological concentrations of their co-agonists glutamate and glycine^5–9^. Physiological NMDAR internalization is mediated by clathrin/dynamin-dependent endocytosis to Rab5-positive early endosomes^9,10^. Carboxy-terminal motifs in GluN2A/B subunits direct endocytosed NMDARs to recycling endosomes and back to the surface, while conserved sequences near the juxtamembrane region of GluN1 and GluN2 drive receptors toward late endosomes and degradation^8^. NMDAR overactivation triggers endocytic processes in neurons preceding death in cellular models of excitotoxicity^11^ or cerebral ischemia^12^. However, very little is known about how NMDAR overstimulation affects its own internalization. A recent publication has shown the excitotoxic induction of GluN2B-containing NMDARs endocytosis in primary neurons and the relationship of this endocytic process with neuronal death^13^. Despite these data, the molecular mechanisms downstream endocytosis of overactivated NMDARs and their connection to excitotoxicity remain unexplored.

Kinase D-interacting substrate of 220 kDa (Kidins220^14^), also known as ankyrin repeat-rich membrane spanning (ARMS^15^), is an effector of NMDAR signaling essential for neuronal viability by the control of ERK^16,17^. Silencing of Kidins220 reduces basal ERK activity and neuronal survival, additionally potentiating NMDAR-mediated excitotoxicity^16,17^. Transient cerebral ischemia and in vitro excitoxicity decrease Kidins220 levels^16^. This downregulation contributes to neuronal death and primarily depends on Kidins220 rapid cleavage by the Ca^2+^-dependent protease calpain, activated by high Ca^2+^ influx through overactivated NMDARs^16,17^. Remarkably, GluN2 and GluN1 subunits are also downregulated by excitotoxicity^18,19^. Considerable evidences support the impact of downregulation of these components on neuronal survival^16–19^. There are no proofs however of Kidins220 endocytosis induced by NMDAR stimulation or the participation of this internalization in the mechanisms of excitotoxicity.

Here we find that excitotoxicity induces Kidins220 and GluN1 internalization. Kidins220 is targeted to the Golgi apparatus (GA) through a Rab5-positive endosomal compartment at time points when no calpain degradation has occurred yet, indicating that Kidins220 proteolysis might start once at the GA. We also show that Kidins220 excitotoxic endocytosis and subsequent downregulation governs inactivation of the small GTP-ase Rap1 that ultimately controls decreases in ERK activity preceding neuronal death. Furthermore, we identify the molecular mechanisms involved in the excitotoxic shutoff of Kidins220/Rap1/ERKs prosurvival cascade that depends on calpain processing of Rap1-activation complexes.

## Materials and methods

### Materials and chemicals

*N*-Methyl-D-aspartate (NMDA), glycine, cytosine β-D-arabinofuranoside (AraC), poly-L-lysine, L-laminin, cycloheximide (CHX), and Optiprep were acquired from Sigma Co. (St. Louis, MO, USA). Calpain I (μ-calpain) and calpain inhibitor CiIII were acquired from Calbiochem-Merck Bioscience (Darmstadt, Germany), antagonist 2-amino-phosphopentanoic acid (DL-AP5) from Tocris (Bristol, UK) and Rap1 inhibitor GGTI289 from Millipore Corporation (Billerica, MA, USA). Lipofectamine 2000 and Glutamax were purchased from Thermo Fisher Scientific (Rockford, IL, USA). ECL Western Lighting Chemiluminesence Reagent Plus was purchased from Perkin-Elmer Life Sciences (Boston, MA, USA), and the BCA reagent and Pierce Cell Surface Protein Isolation Kit were purchased from Pierce Thermo Fisher Scientific (Rockford).

### Antibodies

Kidins220 C-terminal rabbit polyclonal antiserum (Kidins220) was generated as described^14^, using as immunogen a 17 amino-acid peptide from the protein C-terminal end. This antibody was used for all immunofluorescence and immunoblot analysis unless otherwise indicated. For immunodetection of Kidins220 in electron microscopy analysis the same purified C-terminal rabbit polyclonal antibody obtained from Abcam (Cambridge, UK) was used. A novel Kidins220 extracellular region (Kid-ER) antibody was generated after rabbit immunization with a peptide (C-LVFAFTVDTNLAIA) containing an extracellular region of the protein (see scheme in Supplementary Fig. 1A). Kid-ER antiserum was purified after protein A-sepharose binding, elution, neutralization, and dialysis by standard procedures before its application for immunofluorescence analysis. The specificity of this new antibody was first validated preabsorbing Kid-ER with the immunizing peptide and performing immunoblot analysis of neuronal lysates (Supplementary Fig. 1B). Kid-ER immunoblot specificity was also confirmed after silencing Kidins220 in cultured neurons (Supplementary Fig. 1C). Control experiments using immunizing peptide-competed antiserum showed Kid-ER immunofluorescence staining was specific (Supplementary Fig. 1D). Rabbit polyclonal antibodies recognizing active phospho-Thr^202^/Tyr^204^ extracellular signal-regulated kinase (pERK-1/2) were from Cell Signaling Technology (Beverly, MA, USA). Rabbit polyclonal antibody recognizing PDZ-GEF1 was generated in N.M.'s laboratory, and those against neuronal specific enolase (NSE) and Rap1 GTP-ase were from ICN Biomedicals (Costa Mesa, CA, USA) and Millipore Corporation (Billerica, MA, USA), respectively. Rabbit polyclonal antibody recognizing H-, K-, and N-Ras and monoclonal antibodies against Shc and Golgi matrix protein of 130 kDa (GM130) were acquired from BD Transduction Laboratories (San Jose, CA, USA). Monoclonal antibodies against β-actin and spectrin, and rabbit polyclonal antibody recognizing α-, β-, and γ-isoforms of S-SCAM were purchased from Sigma Co. (St. Louis, MO, USA). Mouse monoclonal antibodies specific for α−1, β−1, and β−2 isoforms of syntrophin, GluN1, and hemagglutinin epitope were purchased from Affinity BioReagents (Golden, CO, USA), Pharmingen (San Diego, CA, USA) and Covance (Berkeley, CA, USA), respectively. Rabbit polyclonal antibodies recognizing total ERK-1/2, C3G, Grb2, SOS, Crk, and FRS2 and were purchased from Santa Cruz Biotechnology (Santa Cruz, CA, USA). Horseradish peroxidase-conjugated and Alexa-Fluor-488, -546, and -647-coupled antibodies were purchased from General Electric (Fairfield, CT, USA) and Molecular Probes (Thermo Fisher Scientific; Rockford, IL, USA).

### Cell culture and treatment of primary cortical neurons

Neuronal cultures were prepared from cerebral cortex of 19-day-old Wistar rat embryos, as we have previously described^16,19^. Rats were obtained from the animal care facility at the Instituto de Investigaciones Biomédicas “Alberto Sols” (CSIC-UAM, Madrid, Spain). Animal procedures were approved by the ethical committee from the CSIC and performed in compliance with European Directive 2010/63/EU. Neurons were used after 10 days in vitro (DIV) for immunofluorescence and DIV14 for biochemical assays. Neurons were pretreated or treated for different times as indicated with the following concentrations of reactives: 100 μM NMDA, 10 μM glycine, 200 μM DL-AP5, 10 μM CiIII, 200 μM CHX, 15 μM Lactacystin, 100 μM zVAD, and 10 μM GGTI298. Excitotoxicity was induced by treatment with the NMDAR co-agonists NMDA and glycine. Unless otherwise stated inhibitors were added 1 h before treatment with NMDA/glycine and remained in the culture media for the duration of the experiment.

### Preparation of protein extracts, immunoblot analysis, and immunoprecipitation

Preparation of protein extracts and immunoprecipitation were performed as described^16^. Equal amounts of total lysates or equivalent volumes of immunocomplexes were resolved on SDS-PAGE and analyzed by immunoblot. Membranes were incubated with different primary and secondary antibodies and immunoreactive bands were detected by ECL (Perkin-Elmer Life Sciences, Boston, MA, USA).

### Plasmids and transfection of neurons

Golgi-GFP, Rab5-GFP, and HA-Rap1 constructs were kindly provided by Professors M. Zerial (Max Panck Institute of Molecular Cell Biology and Genetics, Dresden, Germany), V. Malhotra (Centre for Genomic Regulation, Barcelona, Spain), and P. Crespo (Instituto de Biomedicina y Biotecnología de Cantabria, Santander, Spain), respectively. Primary cortical neurons DIV8 were transfected with Lipofectamine 2000 reagent. DNA–liposome complexes were prepared in Neurobasal Medium (Thermo Fisher Scientific; Rockford, IL, USA) and added to the cultures. Two hours later, liposomes were removed, and neurons were fed with conditioned medium and maintained in culture for 48 h before experimental treatments.

### Cloning Rap1A mutants in a lentiviral vector for neurospecific expression

Lentiviral vectors for neurospecific expression of HA-Rap1A and HA-Rap1A-V12 were generated from their corresponding pMT2HA-Rap1A and pMT2HA-Rap1A-V12 plasmids as follows. Both Rap1A-tagged sequences were amplified using forward (5′-CGC **GGA TCC** TTA ATG GCT TAC CCA TAC GAT GT-3′) and reverse (5′-CAT TAA **GCG GCC G**CC ACT TTC CAA TTA GGC AAC A-3′) primers containing BamH1 and Not1 restriction sites, respectively (bold). Once digested, PCR products were subcloned in a lentiviral vector bearing human *Synapsin* promoter (*SYN*pr) SYNpr-DsRed-SYNpr-GFP^20^, where DsRed sequence had been previously depleted after digestion with BamHI and NotI. Constructs were sequenced using an Applied Biosystems automated DNA sequencer (Thermo Fisher Scientific; Rockford, IL, USA).

### Lentiviral infection of neuronal cultures

Lentiviral transduction was performed as previously described, adding lentiviral particles to the growing media of DIV8 neurons and left for 6 additional days^16^.

### Cell surface protein biotinylation assays

DIV10 untreated neurons or stimulated with NMDA for the indicated times were placed on ice, washed two times with ice-cold PBS containing 1 mM CaCl_2_ and 0.5 mM MgCl_2_ (PBS/Ca^2+^/Mg^2+^), and surface biotinylated with the Pierce Cell Surface Protein Isolation Kit (Pierce, Thermo Fisher Scientific; Rockford, IL, USA). Briefly, EZ-Link Sulfo-NHS-SS-Biotin was freshly dissolved at 0.7 mg/ml in ice-cold PBS/Ca^2+^/Mg^2+^ and added to the cultures. Incubation proceeded for 12 min at 4 °C with constant shaking. Then, biotin solution was removed and dishes were blocked by washing three times with PBS/Ca^2+^/Mg^2+^ containing 0.1% BSA and two times with ice-cold PBS/Ca^2+^/Mg^2+^ to eliminate the excess of biotin reagent that was not efficiently incorporated to the cell surface. Finally neurons were lysed in RIPA buffer containing protease and phosphatase inhibitors^16^, and solubilised by rotating 1 h at 4 °C. Nuclear and cellular debris were removed by centrifugation at 12,000 rpm for 5 min at 4 °C and supernatants were precipitated with 100 μl of UltraLink NeutrAvidin slurry (Pierce, Thermo Fisher Scientific) for 2 h at 4 °C. Beads were washed twice in RIPA buffer containing 500 mM NaCl and once in RIPA buffer containing 150 mM NaCl. Beads were resuspended in 100 μl of sample buffer and resolved on SDS-PAGE.

### Internalization of biotinylated surface proteins

To analyze internalization of cell surface proteins, untreated DIV10 neurons were placed on ice and washed twice with ice-cold PBS/Ca^2+^/Mg^2+^. Biotin was added as explained for cell surface biotinylation assays, left for 15 min at 4 °C, removed and cultures were blocked with ice-cold PBS/Ca^2+^/Mg^2+^ containing 0.1% BSA. After washing unbound biotin twice with PBS/Ca^2+^/Mg^2+^, conditioned growth medium was added back to neurons that were returned to the incubator for 30 min at 37 °C. Cultures were then treated with NMDA for 10 min or left unstimulated. Biotin surface cleavage was performed by placing the dishes on ice and incubating with ice-cold cleaving buffer (50 mM glutathione in 75 mM NaCl, 10 mM EDTA, 1% BSA, and 0.075 M NaOH) twice for 10 min at 4 °C with constant shaking. Finally neurons were washed four times in ice-cold PBS/Ca^2+^/Mg^2+^, lysed in RIPA buffer and supernatants were precipitated with UltraLink NeutrAvidin beads for immunoblot analysis as indicated above for cell surface biotinylation assays.

### Immunofluorescence and confocal microscopy

For immunofluorescence neurons grown on coverslips were fixed for 15 min in 4% paraformaldehyde in PBS containing 4% sucrose at 37 °C. After blocking, cells were incubated at room temperature for 2 h with the corresponding antibody diluted in 0.05% saponin in PBS. Immunoreactivity was detected with suitable fluorophore-conjugated secondary antibody before mounting on slides with Prolong (Thermo Fisher Scientific; Rockford, IL, USA). Images are single sections of a *z*-series acquiring each channel in a sequential mode using an inverted Zeiss LSM710 confocal microscope with a ×63/1.40 Plan-Apochromatic objective. Pictures were processed with ZEN 2009 light Edition (Carl Zeiss MicroImaging) and Adobe CS3 Extended (Adobe Systems Inc.) software. Pearson correlation coefficient (PCC) was assed using ImageJ (v1.47d, NIH, Bethesda, MD, USA).

For surface localization, DIV10 cortical neurons were treated with NMDA for 30 min, immediately fixed in 4% paraformaldehyde in PBS at 4 °C for 4 min. Blocking with 2% BSA and 2% donkey serum for 30 min was followed by incubation with Kid-ER overnight at 4 °C, application of secondary antibody and mounting as above. Quantification of fluorescence intensity was carried out using ImageJ software. Neurons were thresholded by gray value at a level close to 50% of the dynamic range. Background noise from these images was negligible. Average fluorescence was quantified after selecting a dendrite segment in the differential interference contrast image of untreated or NMDA-treated neurons.

### Immunoelectron microscopy and quantification

Subcellular localization of Kidins220 in cortical cultures in control conditions and after treatment with NMDA for 10, 30, and 60 min was analyzed using the preembedding immunogold method^21^. Briefly, DIV10 neurons were fixed using 4% paraformaldehyde plus 0.2% glutaraldehyde plus 15% picric acid in 0.1 phosphate buffer (PB), pH7.4, during 4 h. Cultures were then incubated at room temperature for 1 h in 10% normal goat serum (NGS) diluted in TBS and for 24 h in anti-Kidins220 antibody (Abcam, Cambridge, UK) at a final protein concentration of 1–2 μg/ml diluted in TBS containing 1% NGS. After several washes in TBS, sections were incubated for 3 h in goat anti-rabbit IgG coupled to 1.4 nm gold (Nanoprobes) diluted 1:100 in TBS containing 1% NGS. The cultures were then washed in PBS and postfixed in 1% glutaraldehyde diluted in the same buffer for 10 min. They were washed in double-distilled water, followed by silver enhancement of the gold particles with an HQ silver kit (Nanoprobes). This processing was followed by treatment with osmium tetraoxide (1% in 0.1 M PB), block-staining with uranyl acetate, dehydration in graded series of ethanol, and embedding in Durcupan (Fluka) resin. Cortical cultures were cut at 70–90 nm on an ultramicrotome (Reichert Ultracut E; Leica) and collected on 200-mesh nickel grids. Staining was performed on drops of 1% aqueous uranyl acetate followed by Reynolds’s lead citrate. Ultrastructural analyses were performed in a Jeol-1010 electron microscope. Immunogold labeling was evaluated quantitatively to test differences in Kidins220 distribution in control conditions and after treatment with NMDA. Quantification was performed on nine ultrathin sections randomly chosen from each of the two different coverslips. Electron microscopic serial ultrathin sections were cut close to the surface of each block. Randomly selected areas were captured at a final magnification of ×40,000 for each experimental group. In each reference area, the numbers of gold particles attached to the plasma membrane of cell bodies and neurites, and membranous intracellular compartments were counted. Then the percentage of immunoparticles positive for Kidins220 was calculated in control conditions and after treatment with NMDA. The background labeling was evaluated in the same way calculating the density of Kidins220 over mitochondria, nuclei, and empty resin.

### Subcellular fractionation

Subcellular fractionation was performed using Optiprep (60% iodixanol; Axis Shield PoC AS, Oslo, Norway) density gradient centrifugation following the protocol described by Arevalo et al.^22^ with slight modifications. Cortical neurons (DIV14) were cracked in breaking buffer (250 mM sucrose, 10 mM HEPES pH 7.4, 1 mM EDTA, 1 mM MgOAc and protease and phosphatase inhibitors). A cell homogenizer (Isobiotec Heidelberg, Germany) of 0.5 ml volume was used with a tungsten carbide ball of 7.988 mm diameter. Samples were centrifuged at 3,000 rpm for 5 min at 4 °C, and the obtained postnuclear supernatant was then subjected to a second centrifugation for 30 min at 13,900 rpm at 4 °C to get the membrane fraction. Membranes were resuspended in 50 mM Tris-HCl pH 7.5 and adjusted to 25% iodixanol, loaded at the bottom of a SW40Ti ultracentrifuge tube and overlaid with 20%, 15%, 10%, and 5% iodixanol, all of them prepared in 50 mM Tris-HCl pH 7.5 containing protease and phosphatase inhibitors. Gradients were centrifuged at 27,000 rpm for 18 h at 4 °C using a SW40Ti rotor (Beckman, Fullerton, CA). After gradient centrifugation, 25 fractions were collected from top to bottom of the tube, and equal volumes from each fraction were analyzed by immunoblot.

### Pull-down assays

Rap1-GTP pull-down assays were performed using the Rap1 Activation Assay Kit (Millipore Corporation; Billerica, MA, USA) according manufacture’s specifications. Briefly, untreated neurons or stimulated with NMDA for the indicated periods of time were washed in TBS and lysed in RBL buffer (50 mM Tris.HCl, pH 7.4, 500 mM NaCl, 1% NP40, 2.5 mM MgCl_2_, and 5% glycerol) containing protease inhibitors. The samples were centrifuged for 5 min at 4 °C and 14,000 rpm and the supernatant was collected and incubated for 1 h at 4 °C with 20 μg GST-RalGDS-RBD immobilized on glutathione-sepharose beads. Beads were washed three times with RLB and resuspended in Laemmli reducing sample buffer, and GTP-Rap1 was detected by immunobloting with an anti-Rap1 polyclonal antibody. Ras-GTP pull-down assays were performed as above but using GST-Raf1-RBD immobilized on glutathione-sepharose beads and detection with a pan-Ras antibody.

### Quantitative and statistical analysis

Immunoblot signals were quantified by densitometric analysis (NIH Image), normalized using NSE and expressed relative to values obtained in their respective controls. Results are shown as mean ± standard error of the mean of 3–8 independent experiments. Statistical significance was determined by unpaired Student’s *t*-test. A *p*-value smaller than 0.05 was considered statistically significant: *p < 0.05, **p < 0.01, ***p < 0.001.

## Results

### Kidins220 and GluN1 are internalized early during excitotoxicity through mechanisms dependent on NMDAR overactivation

**Fig. 1:**
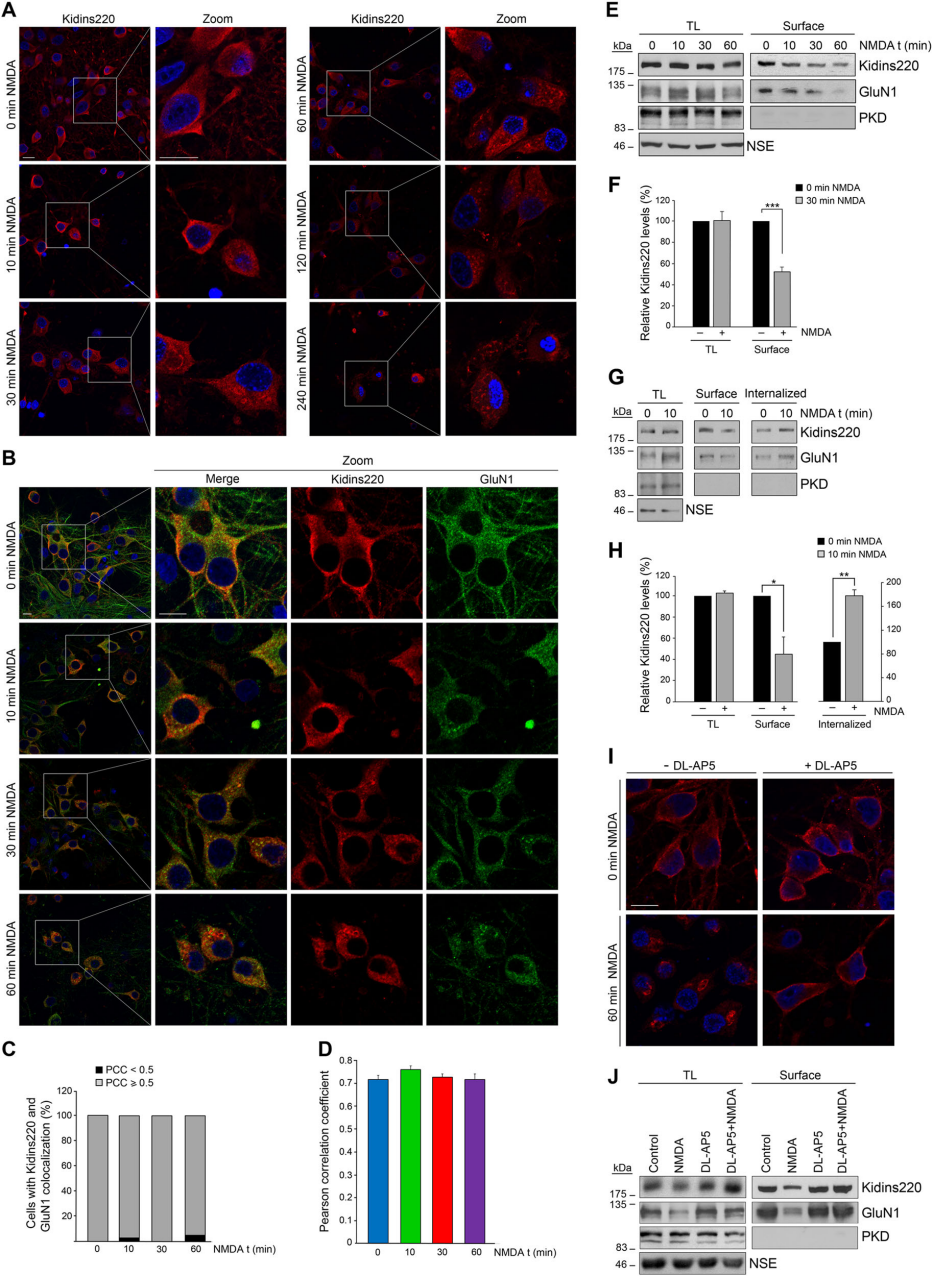
Internalization of Kidins220 and GluN1 by NMDA-induced excitotoxicity. **a** Immunofluorescence of Kidins220 in cortical neurons left untreated or treated with high concentrations of NMDA (100 µM) and the co-agonist glycine (10 µM) (from now referred as NMDA) for the indicated times. Nuclei were stained with DAPI. Merge confocal microscopy images and magnifications corresponding to the boxed areas are depicted. Scale bar: 10 μm. **b** Kidins220 and GluN1 colocalization in control and NMDA-stimulated neurons analyzed by immunostaining and confocal microscopy. Scale bars: 10 μm. **c, d** Percentage of cells displaying colocalization and mean values of Pearson correlation coefficient (PCC), respectively, calculated in single optical sections to quantify Kidins220 and GluN1 colocalization. Data shown are the means ± s.e.m of three independent experiments after counting *n* = 20–30 neurons. **e** Cultured cortical neurons were treated with NMDA for the indicated times and then surface proteins labeled with biotin. The abundance of Kidins220 and GluN1 in total lysates (TL) and in the cell surface was determined by immunoblot. Cytosolic Protein kinase D (PKD) inmunoblot signal was used as a negative control of surface proteins and neuronal specific enolase (NSE) as loading control. **f** Quantification of the effect of 30 min NMDA treatment on Kidins220 surface localization. Data represented are the means ± s.e.m of four independent experiments, and are expressed relative to values obtained in nonstimulated cells arbitrary assigned a value of 100%. ***p < 0.001, Student’s *t*-test. **g** To analyze internalization, surface proteins of cultured cortical neurons were labeled before addition of NMDA for 10 min. The amount of Kidins220 and GluN1 in TL or internalized after cleavage of surface biotin, was assessed by immunoblot. As a control, cell surface labeling at this NMDA time point was performed as in **e**, processed and quantified in parallel. **h** Quantification of the effect of 10 min NMDA treatment on Kidins220 total levels, surface localization, and internalization. Data represented are the means ± s.e.m of three independent experiments, and are expressed relative to values obtained in nonstimulated cells arbitrary assigned a value of 100%. *p < 0.05, **p < 0.01, Student’s *t*-test. **i, j** Confocal microscopy images, surface biotinylation assays, and immunoblot analysis to study dependence of Kidins220 location on NMDAR overactivation. Neurons were preincubated with NMDAR noncompetitive antagonist DL-AP5 (200 μM) before NMDA stimulation for 60 min. Representative results out of three independent experiments are shown. Scale bar: 10 μm

The effects of excitotoxicity on endocytosis of NMDARs and associated proteins, including Kidins220^16^, are largely unknown. We therefore tested whether excitotoxicity could induce Kidins220 internalization. Cultured cortical neurons were exposed to excitotoxic concentrations of NMDA (100 μM) and the co-agonist glycine (10 μM), a treatment referred as “NMDA”. In untreated neurons, endogenous Kidins220 immunostaining showed a more intense signal at the periphery and vesicles of neuronal somas and extensions (Fig. 1a). NMDA treatment changed Kidins220 localization, appearing after 30 min of stimulation along vesicular and semicircular structures reminiscent of membranous intracellular compartments. At later times Kidins220 intensity decreased gradually, concentrated closer to the nucleus with a highly spotted pattern (Fig. 1a). Analysis of GluN1, the obligatory NMDAR subunit^1^, revealed a highly vesiculated staining partially overlapping that of Kidins220 at the cell surface in untreated neurons, as previously observed^16^. Excitotoxicity induced GluN1 redistribution and accumulation with Kidins220 in intracellular membranous structures (Fig. 1b). PCC analysis revealed that 95% of neurons displayed values ≥0.5 at 1 h of treatment, demonstrating a high degree of colocalization (Fig. 1c, d).

Next, we labeled plasma membrane proteins by surface biotinylation and compared levels of biotinylated Kidins220 to total protein in the whole lysates. Specific isolation of surface proteins was assessed by the lack of signal for cytosolic protein kinase D (Fig. 1e). NMDA treatment reduced Kidins220 at the cell surface from 10 min (Fig. 1e), finding a 48 ± 4% decrease upon 30 min of NMDAR overactivation, while corresponding total levels remained unchanged (Fig. 1e, f). Surface GluN1 decreased with similar kinetics (Fig. 1e). The decrease of Kidins220 and GluN1 surface content was not due to downregulation induced by excitotoxicity^16,19^, since this decline occurs later in time. Indeed, incubation with NMDA for 60 min was required to produce a slight reduction on GluN1 and Kidins220 in total lysates (Fig. 1e).

To further confirm Kidin220 internalization, we labeled cell-surface proteins with biotin before 10 min NMDA stimulation. Then, we cleaved biotin from surface proteins and checked internalized biotinylated Kidins220 and GluN1. Surface biotinylated Kidins220 and GluN1 decreased by NMDA treatment while internalized proteins increased (Fig. 1g). For Kidins220, this short NMDA treatment induced a 45 ± 4% decrease in surface levels and a 178 ± 10% increase in the internalized protein (Fig. 1h).

Next, we preincubated neurons with the selective NMDAR antagonist DL-AP5 before adding NMDA for 1 h. Immunostaining and biotinylation assays demonstrated that Kidins220 internalization depended on NMDAR overactivation (Fig. 1i, j). GluN1 internalization was also hampered by DL-AP5 pretreatment. Thus, excitotoxicity induces internalization of NMDAR-complexes containing Kidins220 from the plasma membrane to intracellular compartments.

### Excitotoxicity depletes Kidins220 from the plasma membrane and mediates its recruitment towards the Golgi apparatus

**Fig. 2:**
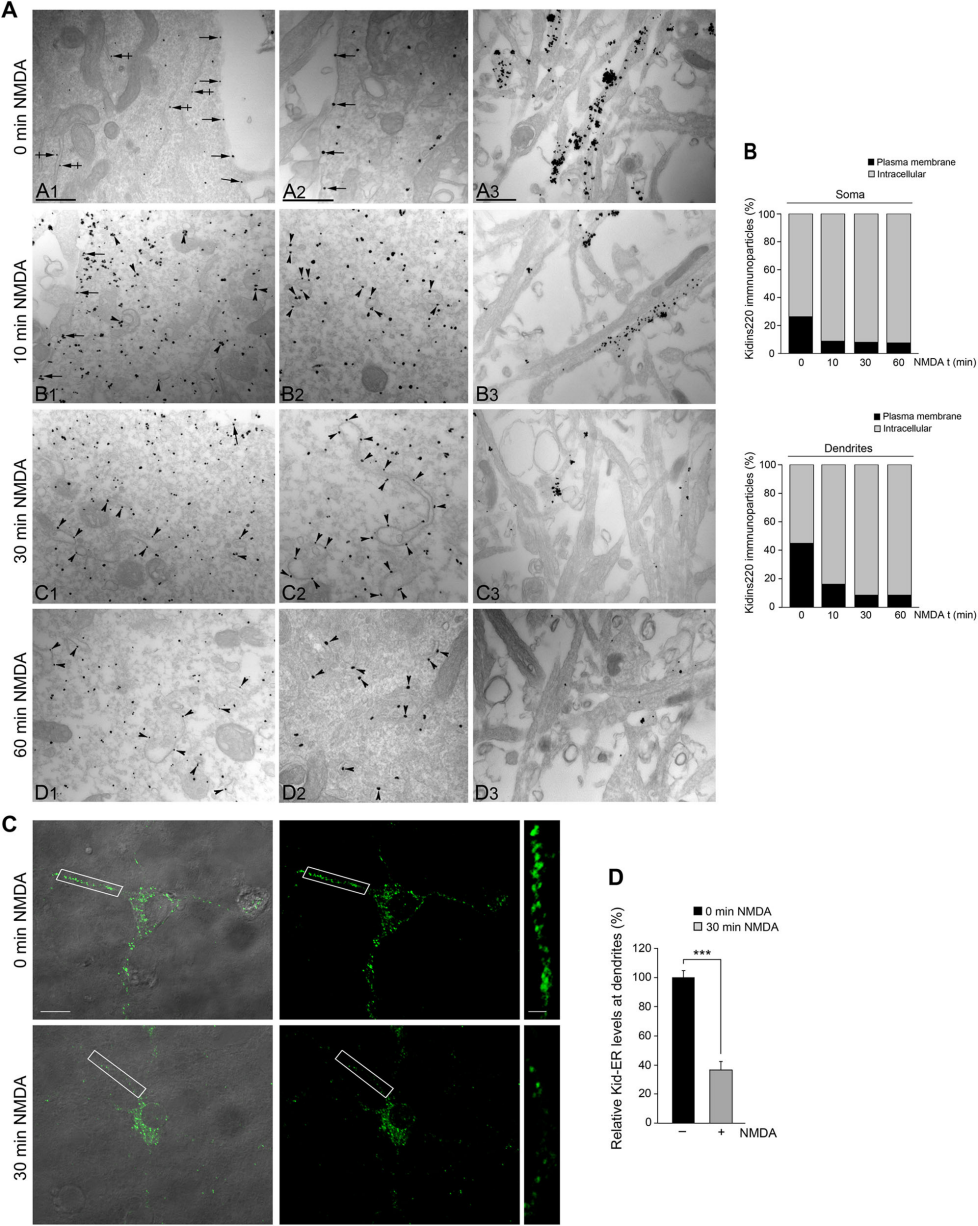
Kidins220 is reduced at the dendritic compartment and enriched in somatic intracellular compartments after NMDARs overstimulation. **a** Kidins220 subcellular localization was analyzed in neuronal cultures untreated (A1–A3) and treated with NMDA for different periods of time (B1–B3, C1–C3, D1–D3) by electron microscopy using a preembedding immunogold method. Representative images are shown. Panels named with subscripts 1 and 2 show immunoparticles present in cell bodies while subscript 3 is used when dendrites are depicted. Plasma membrane (arrows), rough endoplasmic reticulum (crossed arrows), and intracellular vesicles and Golgi apparatus (both as arrowheads) are indicated. Scale bars: 0.5 μm. **b** Quantification of Kidins220-immunoparticles present at the plasma membrane and intracellular sites along NMDA stimulation, both in soma (Top graph) and dendrites (Bottom graph). Two different coverslips of each condition from two independent experiments were used, and nine ultrathin sections were randomly chosen from each coverslip for quantification purposes. **c** Representative images of differential interference contrast (DIC) microscopy and confocal microscopy immunostainig with a novel antibody recognizing Kidins220 extracellular region (Kid-ER) in control or 30 min NMDA-treated, fixed and nonpermeabilized neurons. White boxes indicate magnified dendritic regions. Scale bars, 10 μm and 3 μm. **d** Quantification of mean intensity of dendritic Kidins220 surface staining in untreated neurons versus those NMDA-treated. Data are represented as means ± s.e.m of three independent experiments after quantifying *n* ≥ 18 neurons for each condition. ***p < 0.001, Student’s *t*-test

To characterize ultrastructurally the compartment/s where Kidins220 was internalized, we performed immunogold labeling and electron microscopy analysis of neurons after NMDAR overstimulation (Fig. 2). Before NMDA addition, Kidins220 was abundant in cell bodies and dendrites, along plasma membrane (arrows) and at intracellular membranous compartments (crossed arrows; Fig. 2a, A1–A3, and Fig. 2b). A high concentration of immunogold particles distributed in patches within neuronal extensions (Fig. 2a, A3). In the cell body, Kidins220 was mainly associated with the rough endoplasmic reticulum (Fig. 2a, A1). Only 10 min after NMDARs overstimulation, Kidins220 localization changed significantly towards intracellular sites (Fig. 2a, B1–B3, and Fig. 2b), decreasing along plasma membrane (Fig. 2a, B1–B2, arrows). In cell bodies, particles accumulated beneath plasma membrane or were associated with intracellular vesicles (Fig. 2a, B1–B2, arrowheads). A dramatic reduction of immunoparticles within dendrites was detected at this time-point (Fig. 2a, B3). After 30 min of treatment, Kidins220 concentrated intracellularly at the soma (Fig. 2a, C1–C3, and Fig. 2b), in vesicles, cisternae, and GA fragments (Fig. 2a, C1–C2, arrowheads), while signal along neuronal extensions was almost lost. Stimulation for 60 min increased this effect (Fig. 2a, D1–D3, and Fig. 2b). These results suggest that excitotoxicity provokes a major internalization of Kidins220 from the somatic plasma membrane and dendritic locations towards the GA.

To further assess Kidins220 decrease at the plasma membrane, we developed an antibody recognizing its extracellular region (Kid-ER, see scheme and validation in Supplementary Fig. 1). Inmunofluorescence of neurons treated for 30 min with NMDA detected a decrease in Kidins220 that was significant at processes (Fig. 2c, d), correlating with results obtained by electron microscopy, and strengthening the notion of Kidins220 severe recruitment from the surface of dendritic compartment to intracellular somatic locations under excitotoxic conditions.

**Fig. 3:**
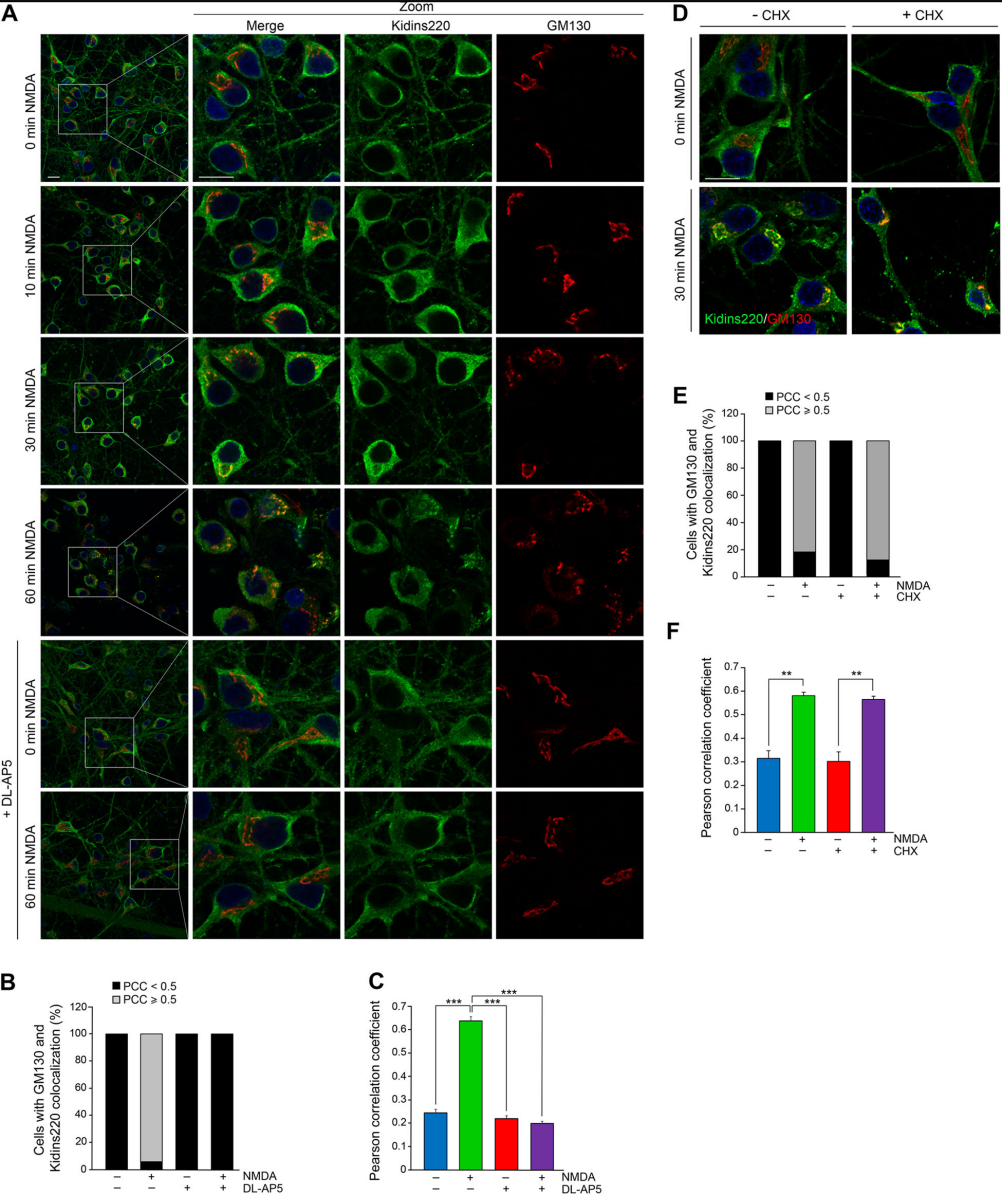
Excitotoxicity induces Kidins220 translocation to the Golgi apparatus. **a** Coimmunostaining of Kidins220 and the Golgi apparatus marker GM130 in neurons treated with NMDA for the indicated times in the absence or presence of the NMDAR antagonist DL-AP5 (200 μM, 60 min). Nuclei were stained with DAPI. Merge confocal microscopy images and magnifications corresponding to the boxed areas are depicted. Scale bar: 10 μm. **b, c** Percentage of cells displaying colocalization of Kidins220 and GM130 and mean Pearson correlation coefficient (PCC) values, respectively, after 60 min of NMDA treatment, calculated in single optical sections obtained from the different conditions. **d** Merged magnified images of Kidins220 and GM130 coimmunofluorescence in neurons preincubated with cycloheximide (CHX; 200 μM, 240 min) before NMDA stimulation for 60 min. **e, f** Percentage of cells displaying colocalization of Kidins220 and GM130 and mean PCC values were calculated as above. Data shown are the means ± s.e.m of three independent experiments after counting *n* = 20–40 neurons. **p < 0.01, ***p < 0.001, Student’s *t*-test

Next, we examined Kidins220 internalization to the GA by confocal microscopy. While Kidins220 was hardly detected at the GA in untreated neurons, its colocalization with the *cis*-Golgi marker GM130 increased significantly after 30 min and 1 h of NMDAR overstimulation (Fig. 3a). No neurons presented a PCC ≥ 0.5 (mean value 0.24 ± 0.02) under basal conditions while, after 60 min of NMDA incubation, 93.4% of neurons showed a PCC ≥ 0.5 (mean value 0.64 ± 0.02) (Fig. 3b, c). Transfection of neurons with the transmembrane domain of Golgi protein mannosidase II fused to GFP (Golgi-GFP) rendered similar results (Supplementary Fig. 2). Excitotoxicity in our in vitro model induced a high degree of GA fragmentation (Fig. 3a), in agreement with other reports^23^. Importantly, preincubation with DL-AP5 prevented Kidins220/GM130 colocalization, as well as fission of Golgi cisternae induced by NMDA (Fig. 3). Preincubation with the inhibitor of de novo protein biosynthesis CHX before NMDA addition for 30 min showed that Kidins220/GM130 colocalization was not affected (Fig. 3d–f). These data indicate that Kidins220 increase at the GA is due to recruitment of previously synthesized protein to this organelle during excitotoxicity. Kidins220 is relocated from the cell surface and other internal compartments to the GA, where it remains recruited during excitotoxic fragmentation of this organelle. Because Kidins220 is detected here by a C-terminal antibody, a region rapidly lost in an excitotoxic- and calpain-dependent manner^16,17^, our results suggest these localization changes take place before its extensive excitotoxic calpain degradation.

### Excitotoxicity increases Kidins220 presence at Rab5-endocytic compartment

**Fig. 4:**
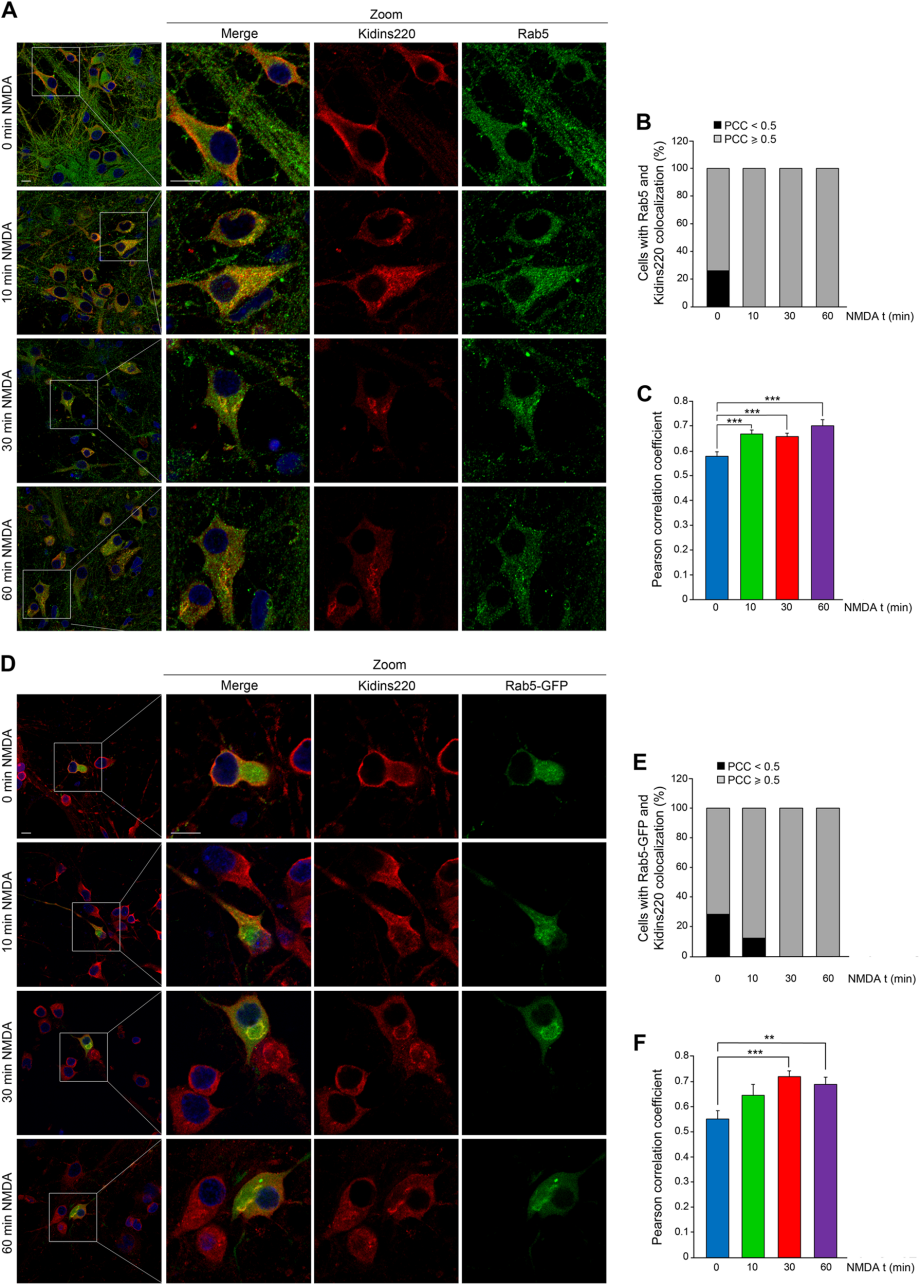
NMDAR overstimulation increases Kidins220 localization at early endosomes. **a** Coimmunostaining of Kidins220 and endogenous Rab5 in neurons treated with NMDA for the indicated times. Nuclei were stained with DAPI. Merge confocal microscopy images and magnifications corresponding to the boxed areas are depicted. Scale bar: 10 μm. **b**, **c** Percentage of cells displaying colocalization of Kidins220 and Rab5 and mean Pearson correlation coefficient (PCC) values, respectively, calculated in single optical sections obtained from the different conditions. Data shown are the means ± s.e.m of three independent experiments after counting *n* = 20–30 neurons. **d** Neurons transfected with Rab5-GFP were treated with NMDA for different times and Kidins220 colocalization was assessed by immunostaining and confocal microscopy. **e**, **f** Colocalization of Kidins220 and Rab5-GFP was evaluated by calculating in single optical sections the percentage of cells displaying colocalization and establishing the mean PCC values. Data shown are the means ± s.e.m of three independent experiments after counting *n* = 10–15 neurons. **p < 0.01, ***p < 0.001, Student’s *t*-test

Targeting of cargos from the plasma membrane to the GA can occur through different endosomal compartments^24^. Physiological internalization of NMDARs is mediated by Rab5, a GTP-ase linked to early endosomes^9^. We explored whether NMDAR overstimulation similarly induced endocytosis of Kidins220 to a Rab5-positive compartment and found that NMDA treatment increased their colocalization (Fig. 4a). PCC values increased from 0.58 ± 0.01 in unstimulated neurons to 0.67 ± 0.01 after only 10 min of NMDA treatment, Rab5 signal still overlapping with that of Kidins220 after 60 min (Fig. 4b, c). These results were confirmed by overstimulating NMDARs in neurons transfected with a Rab5-GFP construct (Fig. 4d). The PCC obtained in basal conditions (0.55 ± 0.023) increased to mean values of 0.65 ± 0.04 and 0.72 ± 0.02 after 10 and 30 min of NMDA treatment, respectively (Fig. 4e, f). These results suggest that Rab5 may participate in Kidins220 traffic from the plasma membrane through early endosomes to the GA during excitotoxicity.

### Excitotoxicity induces Kidins220-mediated Rap1 activation and association to the Golgi apparatus at early times

**Fig. 5:**
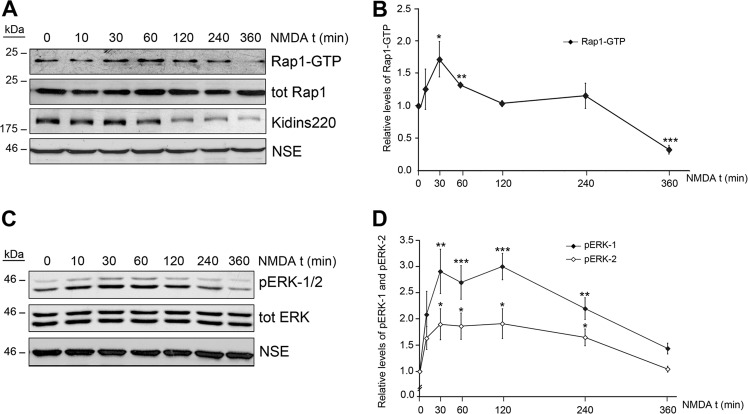
NMDAR overstimulation induces a transient activation of Rap1 and ERK. **a** Rap1 activation curve in response to NMDAR-overstimulation was determined after pulling down active Rap1-GTP with GST-Ral-GDS and immunoblot analysis. **b** The amount of active Rap1-GTP in the pull-down was normalized to total Rap levels present in the lysates and represented relative to the value obtained in nonstimulated cells, arbitrary assigned a value of 1. Results shown are the means ± s.e.m. of five independent experiments. **c** ERK activation was measured by immunoblot analysis of neuronal cultures stimulated with NMDA for various periods of time. **d** Quantitation of phospho-ERK-1 (pERK-1) and phospho-ERK-2 (pERK-2) levels with time of NMDA treatment normalized to those of total ERK. Values are expressed relative to those found in untreated cells, arbitrary assigned a value of 1. Data represented are the means ± s.e.m. of five independent experiments. *p < 0.05, **p < 0.01 and ***p < 0.001, Student’s *t*-test

Kidins220 mediates the activation of Rap1, a GTP-ase involved in ERK stimulation, downstream neurotrophin receptor endocytosis^22,25,26^. Since Kidins220 contributes to early ERK activation after NMDAR overstimulation^16,17^, we wondered whether Rap1 was activated and participated in this process. Pull-down assays from lysates of cultures exposed to excitotoxicity showed a transient Rap1 activation that was maximal between 30 min and 1 h of treatment, and decreased significantly below control levels at 6 h (Fig. 5a, b). Interestingly, Rap1 activity returned to the control levels from 2 to 4 h of NMDA incubation along with a considerable reduction in Kidins220 and phospho-ERK-1/2 (pERK-1/2) (Fig. 5). In fact, complete time-course analysis of ERK-1/2 activity during excitotoxicity revealed a complex activation/inactivation kinetics, similar to that observed for Rap1. NMDAR overstimulation produced a maximum ERK-1/2 activation after 30 min that was maintained for the next 2 h, decreasing progressively later (Fig. 5c, d). We also analyzed Ras activity since it is induced by physiological activation of NMDARs and could be also controlling ERK stimulation^27–30^. In contrast to Rap1, Ras underwent a very rapid excitotoxic inactivation (Supplementary Fig. 3). Altogether, these results suggested Kidins220 participation in the transient activation of Rap1/ERK pathway in excitotoxicity.

**Fig. 6:**
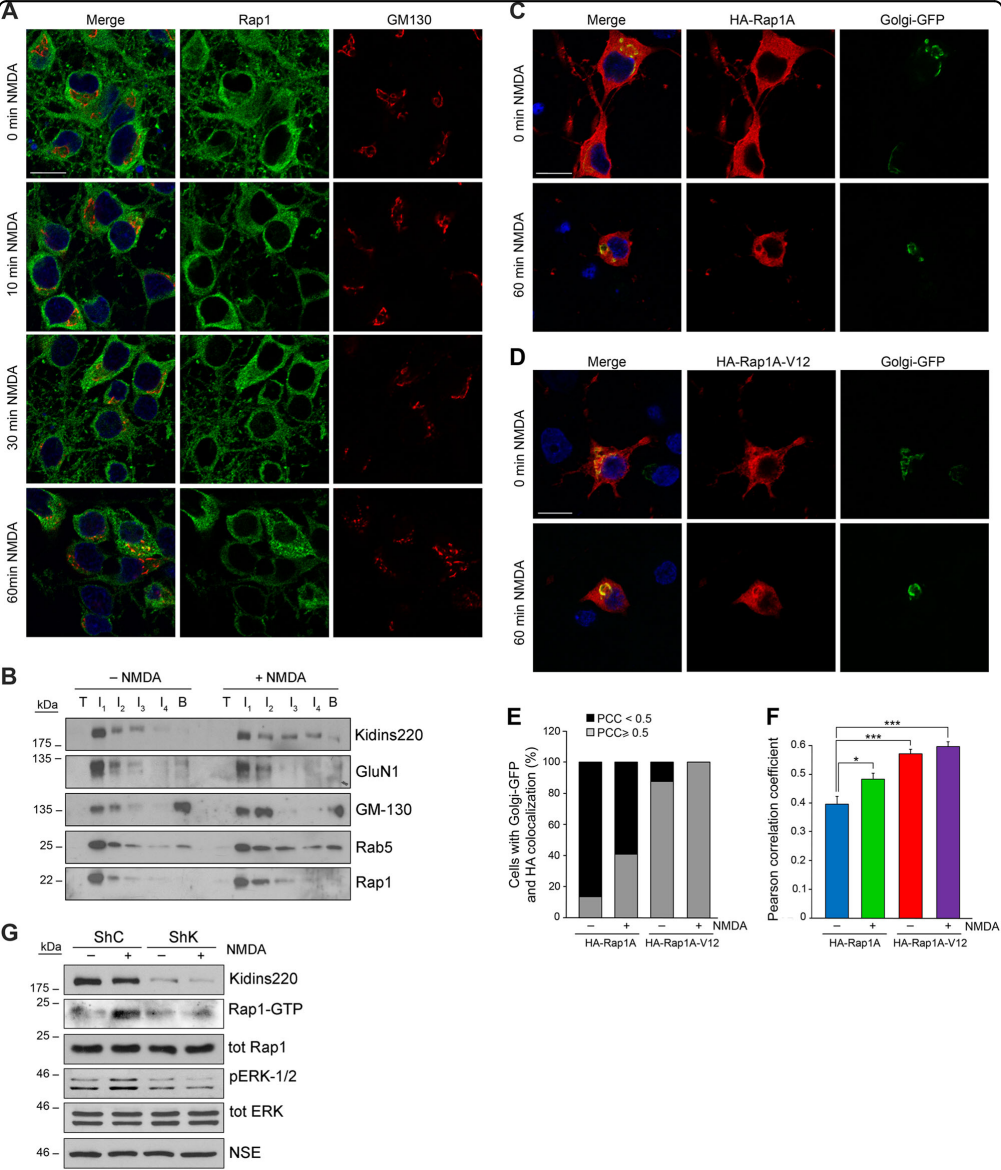
Rap1 translocates to the Golgi apparatus during excitotoxicity and Kidins220 is required for its activation. **a** Endogenous Rap1 and GM130 colocalization in NMDA-stimulated neurons was analyzed by immunostaining. Nuclei were stained with DAPI. Merge confocal microscopy images are shown. Scale bar: 10 μm. **b** Membrane fractions obtained from DIV14 cortical neurons untreated or treated with NMDA for 30 min were subjected to ultracentrifugation in a discontinuous Optiprep gradient. Top and bottom fractions of the gradient, together with the different interfaces (I1–I4), were analyzed by immunoblot with the indicated antibodies. **c**, **d** Neurons were cotransfected with Golgi protein mannosidase II fused to GFP (Golgi-GFP) together with a HA-tagged form of wild-type Rap1A (HA-Rap1A) or its constitutively active form (HA-Rap1A-V12), respectively. The effect of NMDA treatment on the subcellular distribution of transfected Rap1A forms and its overlapping with Golgi-GFP was evaluated by immunofluorescence using an antibody against its HA epitope. Merge confocal microscopy images are shown. Scale bar: 10 μm. **e**, **f** Colocalization of Golgi-GFP and HA-Rap1A or HA-Rap1A-V12 was evaluated by calculating the percentage of cells displaying colocalization in single optical sections and establishing the mean Pearson correlation coefficient (PCC) values. Data shown are the means ± s.e.m of five independent experiments after counting *n* = 10–20 neurons. *p < 0.05 and ***p < 0.001, Student’s *t*-test. **g** Cortical neurons transduced for seven days with lentivirus bearing a shRNA for *Kidins220* silencing (ShK) or control (ShC) were treated for 1 h with NMDA. Rap1 activity was analyzed by pull-down assays and immunobloting. Kidins220 interference, pERK-1/2 and total ERK levels were also detected. Neuronal specific enolase (NSE) was used as loading control. A representative result out of three independent experiments is shown

Because maximal Rap1 activation after NMDAR overstimulation is coincident with Kidins220 internalization to the GA, we hypothesized that Kidins220 could contribute to Rap1 activation at this organelle. Analysis of Rap1 localization showed that it did not localize at the GA in untreated neurons but a partial association was induced after 1 h of NMDA treatment (Fig. 6a). To further study Kidins220 and Rap1 association with early endosomes or GA, we fractionated neuronal membranes. Immunoblot analysis of gradient fractions interfaces (I1–I4) showed Kidins220, GluN1, and Rap1 enriched in I1 together with the endosomal marker Rab5 in control neurons (Fig. 6b). NMDA treatment for 30 min induced a shift of Kidin220, Rap1, and Rab5 towards heavier fractions also containing GM130 (Fig. 6b). Although some GM130 was present in I1 in untreated neurons, excitotoxicity increased its signal in this lighter fraction, together with that in I2, probably indicative of GA initial vesiculation. These results show that excitotoxicity affects Kidins220, Rap1, Rab5, and GM130 mobility in the gradients, provoking their broader distribution and comigration in similar compartments. Together with results in Fig. 5, these data suggested that Rap1 translocation to the GA could be connected to its activation. To explore this possibility, neurons were transfected with HA-Rap1A or a constitutively active mutant HA-Rap1A-V12, together with Golgi-GFP. HA-Rap1A colocalization with Golgi-GFP significantly increased after 1 h of NMDA treatment (Fig. 6c, e, f). However, active HA-Rap1A-V12 already localized at the GA in an 87% of untreated cells reaching a 100% in NMDA-treated neurons (Fig. 6d–f). Endogenous Kidins220 also colocalized with HA-Rap1 or HA-Rap1-V12 at the GA after 1 h of NMDARs overstimulation (Supplementary Fig. 4). Together, these results show that excitotoxicity promotes the association of Rap1 and Kidins220 to the GA, and support that active Rap1 associates to this compartment.

The decrease in Kidins220 levels registered at late excitotoxicity times could contribute to Rap1 inactivation and consequently to that of ERK. To check this hypothesis, we transduced cultured neurons with lentiviruses bearing a shRNA for Kidins220 silencing (ShK) or a control sequence (ShC), and performed pull-down assays to determine Rap1 activity (Fig. 6g). Importantly, Kidins220 silencing blocked NMDA-induced Rap1 activation at 1 h of NMDA treatment, demonstrating that Kidins220 is necessary for an effective activation of Rap1. The absence of Rap1-GTP in ShK transduced neurons was accompanied by reduced levels of p-ERK-1/2, strongly suggesting that excitotoxic activation of Rap1 downstream Kidins220 is governing ERK activity.

### Rap1 regulates ERK activity in excitotoxicity

**Fig. 7:**
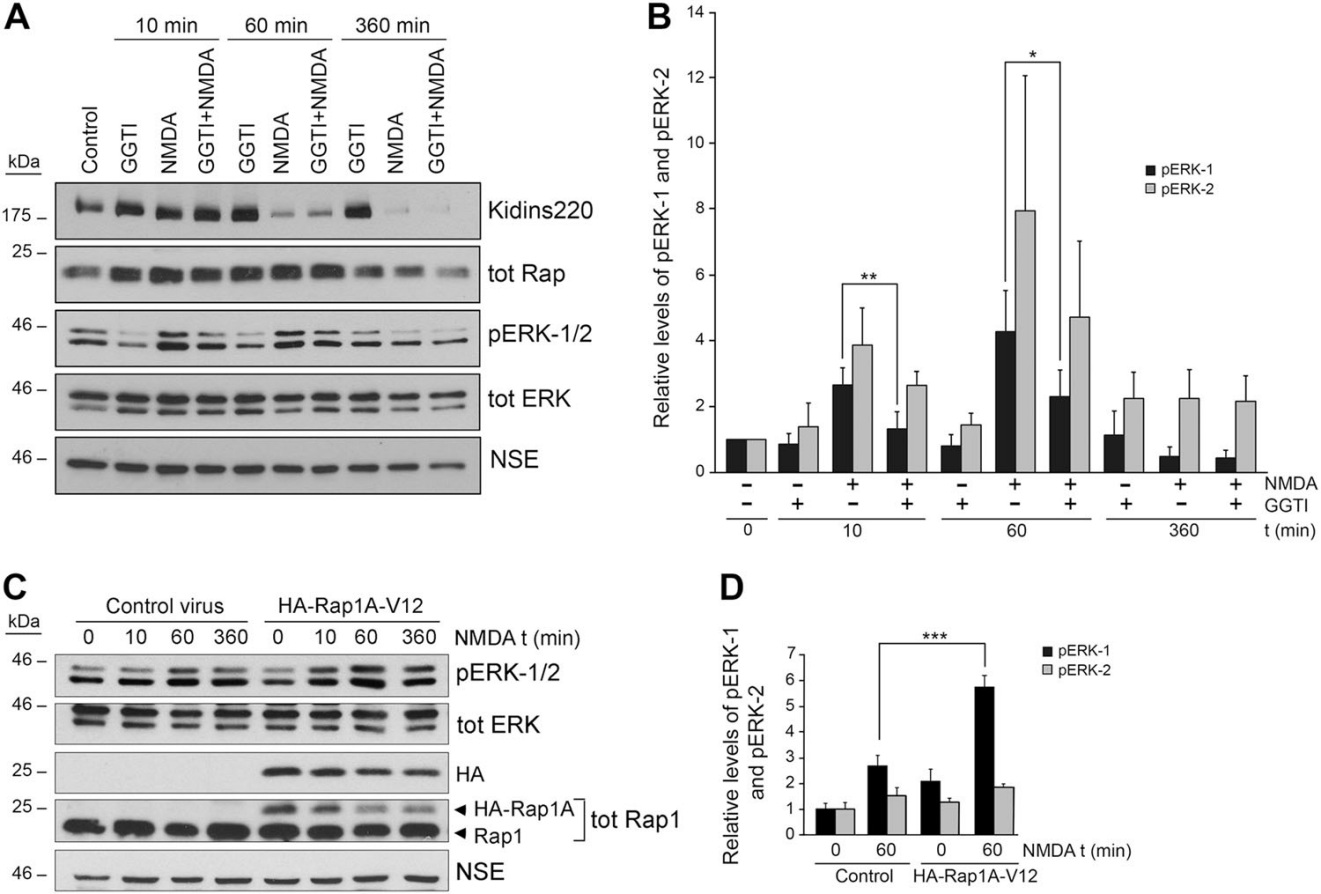
Excitotoxic activation of Rap1 contributes to ERK-1/2 activation. **a** Cortical cultures were incubated 1 h with Rap1 inhibitor GGTI289 (GGTI, 10 μM) prior to NMDA stimulation for the indicated times. Kidins220, Rap1, and ERK-1/2 were analyzed by immunoblotting. **b** Quantification of pERK-1 and pERK-2 levels after normalization with those for total ERK. Results are expressed relative to values found in untreated cells, arbitrary assigned a value of 1. Data represented are the means ± s.e.m. of three independent experiments. **c** Cortical cultures transduced with control or HA-Rap1A-V12 lentiviruses were treated with NMDA for the indicated times and ERK-1/2 activation was assessed by immunoblot. HA, Rap1, and total ERK-1/2 signals were also determined. **d** pERK-1 and pERK-2 levels were normalized to those of total ERK and represented relative to values found in untreated cells, arbitrary assigned a value of 1. Data represented are the means ± s.e.m. of three independent experiments. *p < 0.05, **p < 0.01, and ***p < 0.001, Student’s *t*-test

To establish whether Rap1 may regulate ERK activity during excitotoxicity, we used the Rap1 inhibitor GGTI289 and found that this compound reduced ERK-1/2 activation in response to 10 min and 1 h of NMDA stimulation (Fig. 7a, b). Additionally, we cloned the constitutively active Rap1A mutant (HA-Rap1A-V12) in a lentiviral vector under the control of the human *Synapsin* promoter for its neurospecific expression^20^. Immunoblot analysis of neurons transduced with HA-Rap1A-V12 or control lentivirus showed that constitutive Rap1 activation increased phosphorylated ERK-1/2 in the presence of NMDA at short times of NMDA treatment, and slightly delayed ERK inactivation at later times of excitotoxicity (Fig. 7c, d).

### Calpain-dependent degradation of Kidins220/PDZ-GEF1/S-SCAM Rap1-activation complexes at later times of excitotoxicity

**Fig. 8:**
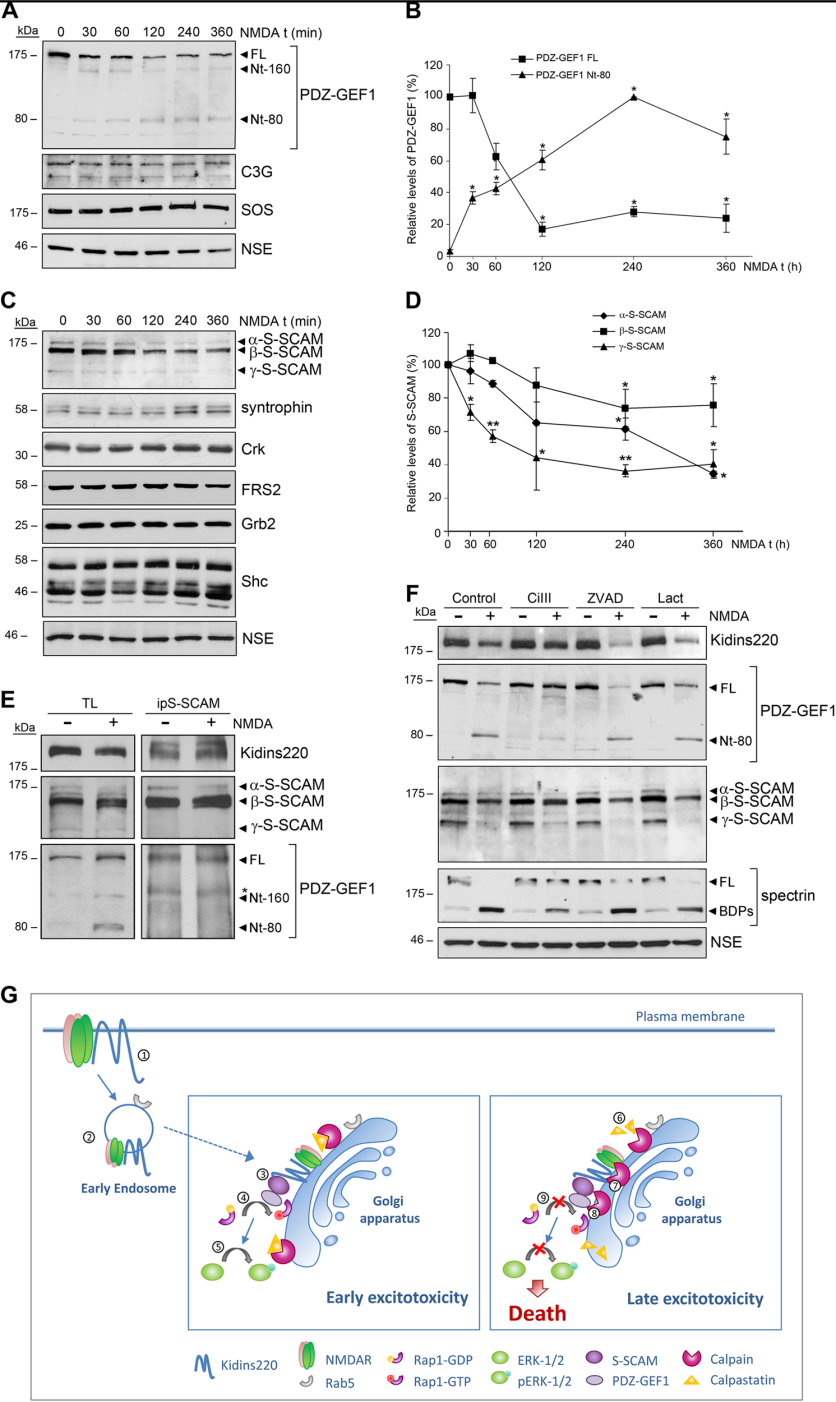
The components of Rap1 activation complexes S-SCAM and PDZ-GEF1 are downregulated during excitotoxicity by calpain-dependent mechanisms. **a** Immunoblot analysis of neuronal cultures stimulated with NMDA for various periods of time using antibodies for PDZ-GEF1 and the indicated proteins. Full-length PDZ-GEF1 (FL) and its derived N-terminal fragments (Nt-160 and Nt-80) are indicated (black arrowheads). **b** Levels of FL and Nt-80 PDZ-GEF1 were normalized to those of NSE and expressed relative to values found in untreated cells, arbitrary assigned a value of 100% (FL) or 0% (Nt-80). Data represented are the means ± s.e.m. of four independent experiments. *p < 0.05, Student’s *t*-test. **c** Immunoblot analysis of neuronal cultures stimulated with NMDA for various periods of time using antibodies for S-SCAM and the indicated proteins. **d** Quantification of different S-SCAM isoforms levels normalized to those of NSE and expressed relative to values found in untreated cells, arbitrary assigned a value of 100%. Data represented are the means ± s.e.m. of four independent experiments. *p < 0.05 and **p < 0.01, Student’s *t*-test. **e** S-SCAM immunoprecipitation (ip) from untreated or 1 h NMDA-treated neurons performed to detect Kidins220, PDZ-GEF1, and S-SCAM association. The asterisk (*) designates a nonspecific band. **f** Immunoblot analysis showing the effect of inhibitors specific for calpain (calpain inhibitor III, CiIII, 20 µM), capases (Z-VAD, 100 µM) or the proteasome (lactacystin, Lact, 15 µM) on PDZ-GEF1 and S-SCAM processing induced in excitotoxicity. Neuronal cultures were incubated with protease inhibitors for 1 h before addition of NMDAR co-agonists. Inhibitors were present for the duration of NMDA treatment. Reduced cleavage of the calpain substrate spectrin into breakdown products (BDPs) demonstrates the efficiency of calpain inhibition. **g** Model of Kidins220 recruitment to the GA and Rap1/ERK activation/inactivation response to excitotoxicity. Kidins220 forms complexes at the neuronal surface with NMDARs (1). At early times of excitotoxicity Kidins220 is targeted to the GA through a Rab5-positive endocytic compartment (2). During this phase, Kidins220 acts as an essential component of Rap1 activation complexes (Kidins220/PDZ-GEF1/S-SCAM) (3), participating in Rap1 activation (4) and the subsequent stimulation of ERK-1/2 (5). At later times of excitotoxicity, the increase in intracellular Ca^2+^ induced by NMDARs overstimulation could lead to partial activation of Golgi-associated calpain, the cleavage of its inhibitor calpastatin (6), and full enhancement of calpain proteolytic activity. Activated calpain will then process cargos such as Kidins220 (7), and other components of Rap1 activation complexes, PDZ-GEF1 and S-SCAM (8) associated to the GA. Thereby, late excitotoxicity turns off Rap1/ERK cascade (9), thus compromising neuronal survival

The temporal coincidence of Kidins220 downregulation and Rap1 inactivation at later times of excitotoxicity drove us to examine the effects of NMDA treatment in Rap1-activation complexes. We analyzed complexes involved in Rap1 activation downstream neurotrophin receptor and Kidins220 signaling, such as the one constituted by the Postsynaptic density-95, Disc Large Zonula occludens (PDZ) proteins S-SCAM, and PDZ-GEF1 or that formed by CrkL/C3G^22,25,26^. The activator of Rap1 PDZ-GEF1 decreased >80% in just 2 h of NMDA treatment (Fig. 8a, b). The simultaneous appearance of two PDZ-GEF1 fragments of 160 and 80 kDa (Nt-160 and Nt-80), recognized by an *N*-terminal antibody, suggested that Nt-80 might generate by Nt-160 proteolysis (Fig. 8a, b). In contrast Rap1 activator C3G and Ras activator SOS were not modified by excitotoxicity (Fig. 8a). Importantly, the different isoforms of the adaptor PDZ-protein S-SCAM (α-S-SCAM, β-S-SCAM, γ-S-SCAM) were progressively downregulated by NMDA (Fig. 8c, d). No response was observed for α-syntrophin (Fig. 8c), another PDZ protein that similarly to S-SCAM interacts with both NMDARs^31–33^ and Kidins220^26,34^, or other adaptor proteins such as Crk and FRS2, or Grb2 and Shc, respectively involved in Rap1^22,25,35–37^ and Ras^38^ activation (Fig. 8c). Antagonist DL-AP5 preincubation confirmed that NMDAR overactivation was involved in PDZ-GEF1 and S-SCAM downregulation (Supplementary Fig. 5A, B). Together, these data demonstrate that excitotoxicity specifically targets Kidins220, PDZ-GEF1 and S-SCAM to degradation, and strongly suggest the existence of Kidins220/PDZ-GEF1/S-SCAM Rap1-activation complexes at early times of excitotoxicity when Rap1 activity is maximum. Co-immunoprecipitation assays confirmed that full-length PDZ-GEF1 was associated with S-SCAM in control and NMDA-treated cells (Fig. 8e), in agreement with previous data describing that these molecules form a binary complex independently of the presence of a stimulus^26,39^. Similarly, Kidins220 was present in S-SCAM immunocomplexes in both conditions, demonstrating that the basal interaction of these proteins is maintained at early times of excitotoxicity (Fig. 8e).

The activity of calpain protease, heavily induced by NMDAR overstimulation, plays a major role in excitotoxicity by processing several substrates^40,41^, including GluN2 subunits^18,42^ and Kidins220^16,17^. By in vitro digestion of neuronal extracts with purified calpain, we established that PDZ-GEF1 and S-SCAM are novel substrates of this protease (Supplementary Fig. 5C, D). We also demonstrated that PDZ-GEF1 and S-SCAM downregulation depends on excitotoxic calpain activation since the specific calpain inhibitor CiIII (10 µM) prevented the decrease of both proteins as it did with Kidins220 (Fig. 8f). Other protease inhibitors such as the pan-caspase inhibitor zVAD (100 µM) or the proteasome inhibitor lactacystin (Lact; 20 µM) exerted no effect (Fig. 8f). These data demonstrate that prolonged excitotoxic stimulation of NMDARs triggers calpain activation and processing of Kidins220, S-SCAM, and PDZ-GEF1, all components of a complex involved in Rap1 activation. The combined downregulation of these proteins would precede and be responsible for Rap1 inactivation, contributing to ERK inactivation and neuronal death (see model in Fig. 8g).

## Discussion

Here we show that excitotoxicity induces the traffic of Kidins220 and GluN1 to the GA through a Rab5-endocytic compartment at early times of excitotoxicity in primary neurons (Fig. 8g). Targeting of Kidins220 to the GA precedes extensive degradation of this molecule by Ca^2+^-dependent calpain activation, which starts to be significant after 2 h of NMDAR overstimulation^16^. These data suggest that Kidins220 excitotoxic proteolysis might take place primarily once it gets to the GA. Among other subcellular locations, calpains are tightly bound to GA membranes facing the cytosol together with their endogenous inhibitor calpastatin^43–45^. Excitotoxic calpain activation has been associated to calpastatin processing^46^. Possibly, calpastatin is GA-bound and inhibits calpain under basal conditions (Fig. 8g). However, intracellular Ca^2+^ increases induced by excitotoxicity could trigger partial calpain activation, sufficient to promote calpastatin degradation and full protease activation, followed by proteolysis of cargos at the GA such as Kidins220. This pathway could serve as a general mechanism for excitotoxic downregulation of other endocytosed cargos. Bringing calpain substrates to where calpain is located constitutes a novel mechanism by which active calpains could reach their substrates.

We find that active forms of Rap1 bind to GA at early time-points of excitotoxicity, in accordance with data describing Rap1 and active Rap1A-V12 association to the GA in nonneuronal cell lines^47–50^ and translocation of Rap1 activator PDZ-GEF1 to perinuclear compartments^51^. We have observed Rap1 maximal activity at time-points when Kidins220 has already been recruited to the GA. Since Rap1 excitotoxic activation depends on Kidins220, our data support that Rap1 gets activated as Kidins220 is recruited to this organelle. At later times of excitotoxicity, calpain activation at the GA would be responsible for Rap1 inactivation through combined proteolysis of the Rap1-activation complex Kidins220/S-SCAM/PDZ-GEF1 (Fig. 8g). Given that excitotoxicity does not downregulate other Rap1-activation complexes (such as C3G, CrkL or FRS2), the cleavage of Kidins220/S-SCAM/PDZ-GEF1 could be considered as a highly specific mechanism promoting Rap1 excitotoxic inactivation.

Downregulation of Rap1 activation complexes by excitotoxicity will be ultimately connected to decreases in ERK activity and neuronal death (Fig. 8g). Physiological stimulation of NMDARs in neurons activates the ERK survival pathway^3,52^. Interestingly, we show here that excitotoxicity initially tends to activate Rap1/ERK cascade in response to NMDAR agonist binding, similarly to physiological conditions. By contrast, excessive NMDAR activation renders the opposite final outcome after Ca^2+^ overload and calpain activation, targeting Rap1/ERK activating components for degradation. It is well accepted that neurons try to re-enter the cell cycle in response to toxic glutamate concentrations, resulting in a frustrated survival mechanism that compromises neuronal viability^53,54^. Indeed, NMDARs overactivation triggers a cell cycle re-entry in neurons that does not proceed and is associated to death^55^. Several evidences support that glutamate, NMDARs, Kidins220, and Rap1/ERK pathways participate in the induction of cell cycle re-entry and proliferation. Glutamate and its receptors facilitate neural stem cell division as well as that of malignant gliomas, a type of tumors where Rap1 and ERK activities promote proliferation^56–59^. By contrast, NMDARs antagonists inhibit ERK signaling and supress cancer growth^60,61^. Accordingly, NMDAR blockade in developing rodent brain leads to apoptotic neuronal death^62,63^. In addition, Kidins220 is important for cellular survival but also for proliferation (reviewed in reference ^64^). Thus, degradation of Kidins220/Rap1/ERK activation complexes in neurons identified herein could also contribute to stop pro-cell division signals triggered through NMDARs during excitotoxicity.

In addition to NMDARs, Kidins220 also interacts with tropomyosin-related kinase (Trk) receptors^15^, being obligatory for neurotrophin-sustained Rap1/ERK activation in neural cells^22,25,26^. Our data support that Kidins220 also controls Rap1/ERK signaling cascade downstream overactivated NMDARs, exerting a conserved role to that played downstream of TrkB. Alternatively, Kidins220 could be an effector of both NMDARs and TrkB in a common pathway preserving neuronal survival. Remarkably, TrkB is also downregulated during excitotoxicity^65,66^. Thus, excitotoxic stimulation of NMDARs would shut off this prosurvival cascade by degrading key activating molecules including NMDARs^18,19^, TrkB^65,66^, Kidins220^16,17^, and other members of the Rap1 activation complexes identified here. Globally, these changes would contribute to excitotoxic neuronal death.

## Supplementary information

Supplementary Figures 1–5
