# Supplementary material for: Excitotoxic targeting of Kidins220 to the Golgi apparatus precedes calpain cleavage of Rap1-activation complexes
Source: Cell Death Dis. 2019 Jul 11;10(7):535. doi: 10.1038/s41419-019-1766-z (PMC6624258; doi:10.1038/s41419-019-1766-z)
Supplement: Supplementary file 1 — Supplementary Figures 1–5 [file 41419_2019_1766_MOESM1_ESM.pdf]

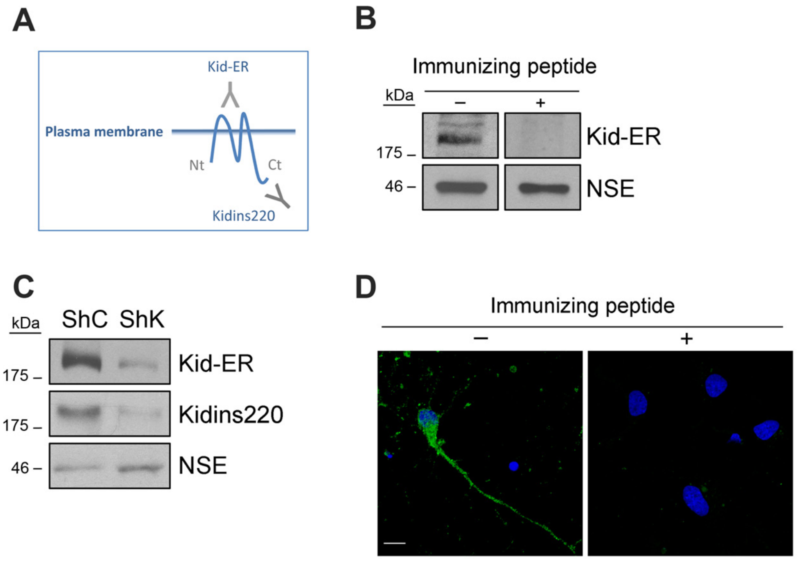

Supplementary Figure 1. Specificity of the antibody recognizing Kidins220 extracellular domain. (A) Scheme showing Kidins220 specific antibodies recognizing the C-terminal (here referred as Kidins220; previously validated[14]) or the extracellular region, indicated as Kid-ER. **(B)** Representative immunoblot of lysates from cultured cortical neurons using Kid-ER. The signal was abolished when pre-incubating the antibody with an excess of the corresponding immunizing peptide (100 μM). **(C)** Representative Kid-ER immunoblot of lysates from neurons transduced with lentiviral particles for Kidins220 silencing (ShK) or control viruses (ShC). Neuronal specific enolase (NSE) was used as loading control and Kidins220 C-terminal antibody as a control of silencing. **(D)** Representative confocal microscopy images of Kid-ER immunofluorescence or that obtained after its competition with the immunizing peptide in cultured cortical neurons. Scale bar 10 μm.

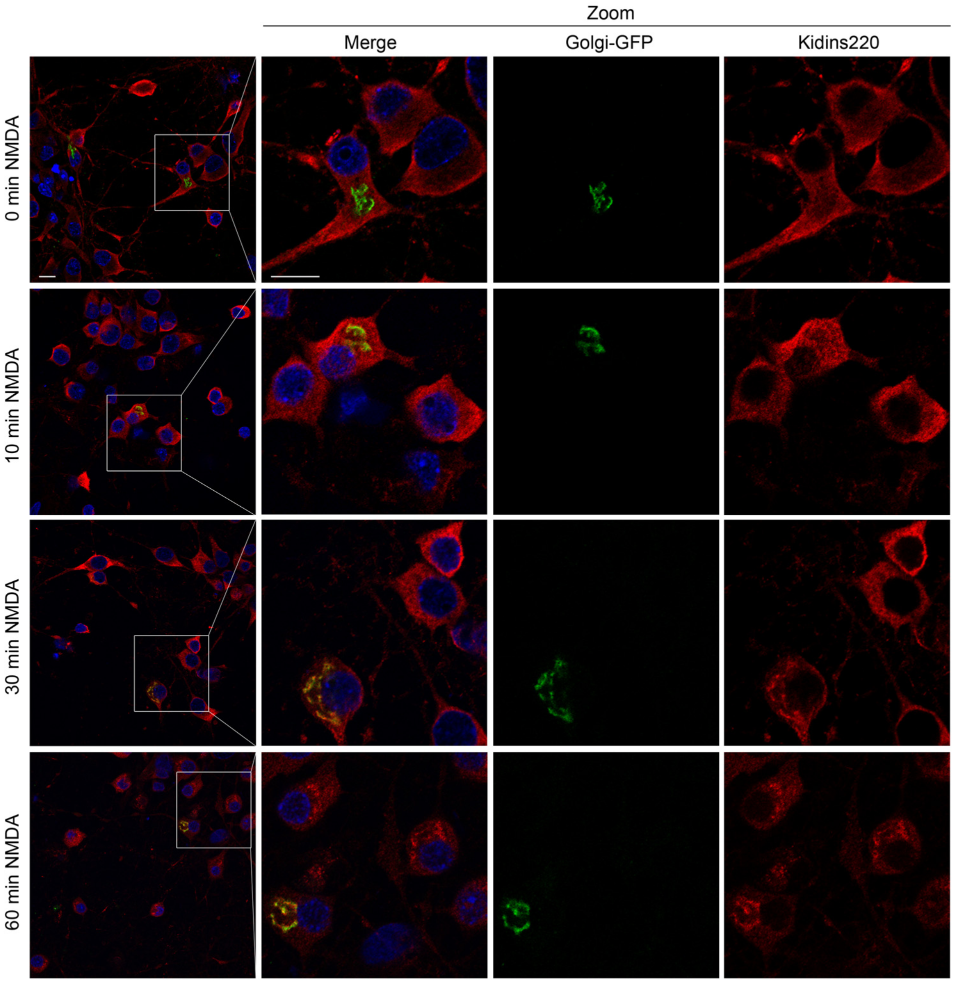

Supplementary Figure 2. K**idins220 colocalises with Golgi-GFP in excitotoxicity.** Neurons transfected with Golgi protein mannosidase II fused to GFP (Golgi-GFP) were treated with NMDA for the indicated times. Kidins220 and Golgi-GFP localization was analysed by immunostaining and confocal microscopy. Nuclei were stained with DAPI. Merge confocal microscopy images and magnifications are shown. Scale bar: 10 µm. A representative result out of three independent experiments is shown.

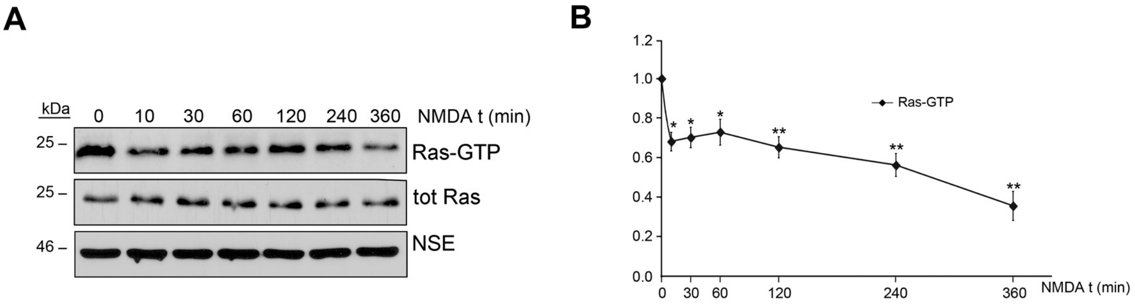

Supplementary Figure 3. Excitotoxic inactivation of Ras. (A) Ras activation curve in response to NMDAR-overstimulation was determined after pulling down active Ras-GTP with GST-Raf-RBD and immunoblot analysis. **(B)** The amount of active Ras-GTP in the pull-down was normalised to total Ras levels present in the lysates and represented relative to the value obtained in non-stimulated cells, arbitrary assigned a value of 1. Results shown are the means ± s.e.m. of five independent experiments. *p<0.05 and **p<0.01, Student's t-test.

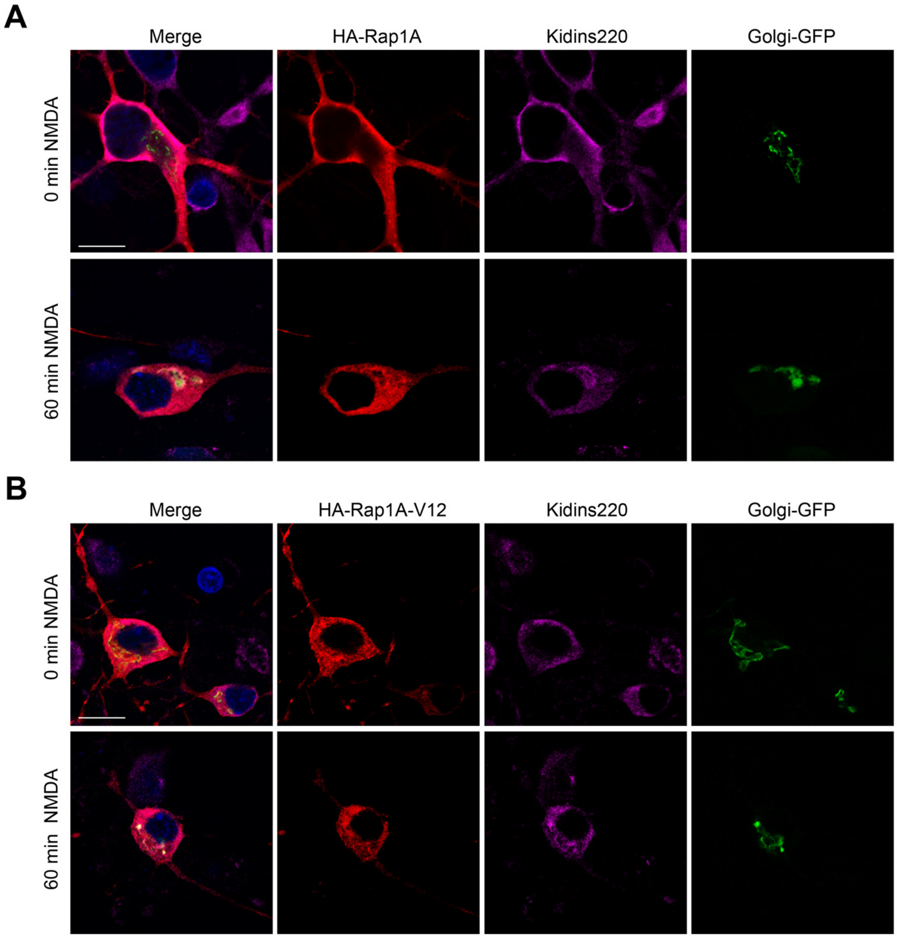

Supplementary Figure 4. Golgi apparatus localization of Kidins220 and active Rap1 under excitotoxic conditions. Neurons transfected with Golgi-GFP and HA-Rap1A **(A)** or its constitutively active form HA-Rap1A-V12 **(B)** were stimulated with NMDA for 1 h. Localization of endogenous Kidins220, Golgi-GFP and HA-Rap1 proteins was determined by immunofluorescence and confocal microscopy. Nuclei were stained with DAPI. Merge confocal microscopy images are shown. Scale bar: 10 μm. A representative result out of three independent experiments is shown.

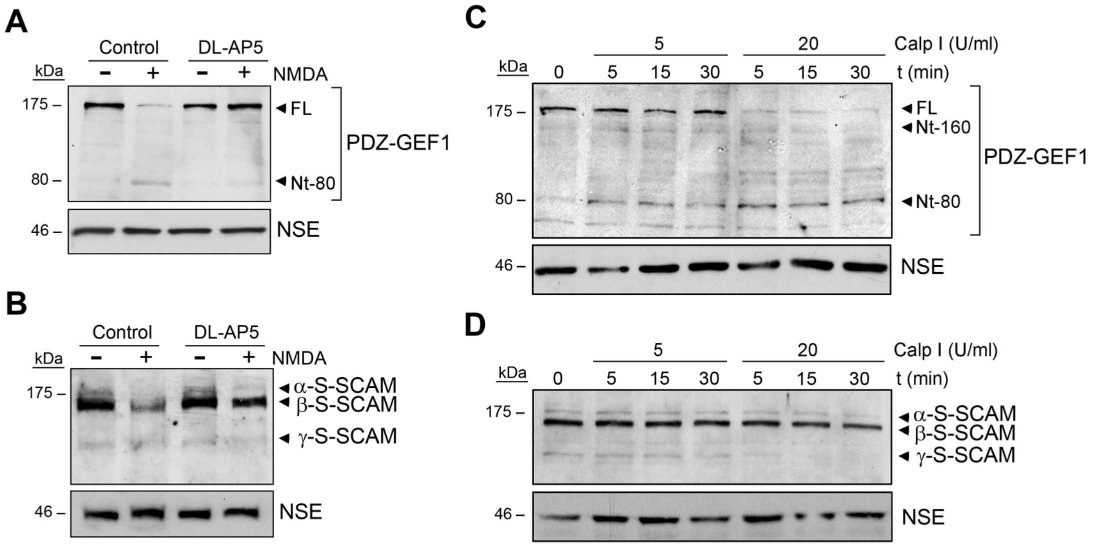

Supplementary Figure 5. PDZ-GEF1 and S-SCAM susceptibility to calpain cleavage. **(A, B)** The dependence of PDZ-GEF1 and S-SCAM downregulation to NMDAR overactivation was assessed by immunoblot. Neuronal cultures were pre-incubated with the non-competitive NMDAR inhibitor DL-AP5 (200 μM) prior to NMDA treatment for 4 h. **(C, D)** Protein extracts from DIV14 neurons were incubated at 37ºC with 0.5 and 20 U/ml of purified calpain I for the indicated time periods. In vitro cleavage of PDZ-GEF1 and S-SCAM was assessed by immunoblot analysis. NSE was used as loading control. A representative result out of three independent experiments is shown.
